# A Retrospective Case Series in Fournier's Disease: And Its Emergency Management et Grafting Technique for Penis Coverage

**DOI:** 10.1155/2022/6710777

**Published:** 2022-09-25

**Authors:** Domenico Tripodi, Antonio Guastafierro, Federica Gagliardi, Maria Ida Amabile, Eleonora Lori, Roberto Cirocchi, Daniele Pironi, Flavio Forte, Claudio Cannistra, Salvatore Sorrenti

**Affiliations:** ^1^Department of Surgical Sciences, “Sapienza” University of Rome, Rome 00161, Italy; ^2^Multidisciplinary Department of Medical-Surgical and Dental Specialties, University “Luigi Vanvitelli” Naples, Naples, Italy; ^3^Department of Surgery and Biomedical Sciences, University of Perugia, Perugia, Italy; ^4^Department of Urology, M.G. Vannini Hospital, Rome, Italy; ^5^Plastic and Reconstructive Surgery Unit, Centre Hospitalier Universitaire Bichat Claude-Bernard, Paris, France

## Abstract

Fournier's gangrene is a necrotizing soft tissue infection of the genital, perineal, and perirectal areas. A primary isolated involvement of the penis is rare, but it can be affected in some circumstances. The purpose of this case series is to present the findings of our thirteen years' experience in the reconstruction of the penis in Fournier's gangrene and our full-thickness grafting technique to cover the penis rod. We retrospectively reviewed patient data who underwent a penis reconstruction following Fournier's gangrene in 2018. The data was analyzed to report the estimated percentage of complications, of patients with primary or secondary gangrene of the penis, the number of reinterventions, and finally the percentage of deaths or recovery. 23 patients underwent reconstruction with our technique of full-thickness skin graft. In all cases, the skin graft was harvested from the upper arm with an arrow shape mark. No further penile revision surgery was required, and neither patient complained about retraction, nor traction, or pain during erection. The donor site healed without any complications. We believe that the coverage of the penis using our grafting technique is safe, easily reproducible, and demonstrates excellent esthetic and functional results.

## 1. Introduction

Fournier's gangrene is a necrotizing soft tissue infection of the genital, perineal, and perirectal areas. [1]. A primary isolated involvement of the penis is rare, but it can be affected in some circumstances [2, 3]. Fournier's gangrene is a life-threatening condition, constituting a urological surgical emergency and has a high mortality rate, ranging from 0 to 67% [4]. Risk factors for the disease are intravenous drug abuse [5], radiotherapy [6], diabetes [7], cancer, and iatrogenic factors [8–10]. Triggers relating to the exclusive penile zone have been identified as the self-injection of Vaseline in the penis' shaft [11], self-introduction of beads in the urethra [12], Phimosis complication [13], penile abrasion from oral sex [3], infection by atypical *Candida* species [14], and Calciphylaxis [15]. Optimal care always requires a multidisciplinary team, the goal being to stabilize the patient with extensive debridement and control the infection with broad-spectrum antibiotic treatment. There are several reconstruction options in this region, and the surgical approach should be tailored to the extent of the residual damage. In the acute phase, aggressive fluid resuscitation, broad-spectrum antibiotics, and immediate radical surgical debridement are required. Secondly, patients will need a definitive reconstruction. Skin grafts and flaps are recommended for reconstruction depending on the situation. The purpose of this case series is to present the findings of our four years' experience in the reconstruction of the penis in Fournier's gangrene and our full-thickness grafting technique to cover the penis rod.

## 2. Materials and Methods

We retrospectively reviewed patient data who underwent a penile reconstruction following Fournier's gangrene in 2018. The collected material included anamnestic clinical data, surgical reconstruction type, surgical timing, median follow-up, and photographic material. The data was analyzed to report the estimated percentage of complications, the number of re-interventions, the percentage of patients with primary or secondary gangrene of the penis, and finally the percentage of deaths or recovery. All patients aged 16 to 59 years with soft tissue defects in the scrotal and penis undergoing tissue covering were included in the study. After discharge from the hospital, patients were followed up weekly in a follow-up clinic for three months taking into account penile size, color, scar appearance, and donor site morbidity.

### 2.1. Medical Therapy

Fournier's gangrene is often caused by a polymicrobial infection that progresses to obliterative endarteritis with microthrombosis along the fascial planes. It begins in the genitals or perineum and spreads further along Buck's fascia, Colle's fascia, and in some cases, Scarpa's fascia. Edema and impaired blood circulation lead to a progressive exponential increase in peripheral dissection with overlying skin and necrosis of the subcutaneous tissue. Due to thrombosis of small vessels and subsequent hypoxia, facultative and obligatory anaerobic bacteria prevail. We found that *Escherichia coli* (27.5%), *Staphylococcus* sp. (12.1%), *Streptococcus* sp. (9.3%), and *Pseudomonas* sp. 6.6%) were the most common causative organisms. Diagnosis of FG can be difficult due to the presentation of nonspecific symptoms. Scrotal swelling, fever, pain, necrosis, and changes in erythema and edema were the most common presenting symptoms. Ultrasound and CT can help in the diagnosis; however, early treatment should not be delayed, they are essential to reduce mortality. Fluid resuscitation should be started immediately, electrolyte and blood glucose imbalances corrected, as poor control can lead to aggressive disease progression. Broad-spectrum therapy, aerobic, anaerobic, and fungal and urine blood cultures can be initiated and once their results are available, they can be selected based on sensitivity and continued until surgical control is achieved depending on the patient.

### 2.2. Surgical Technique

**Figure 1:**
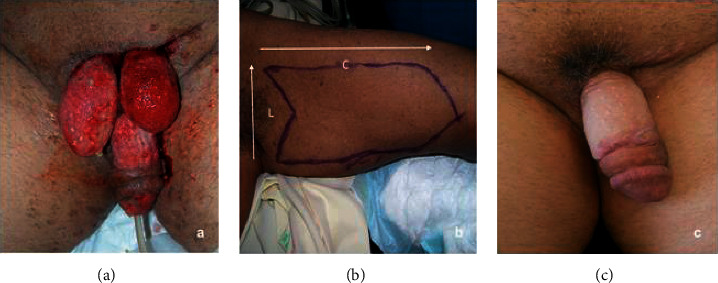
(a) Granulation tissue after debridement and local care. (b) Arrow-shaped drawing on donor skin graft site. (c) Outcomes at 1 year, testicles set in the internal side of the thigh.

**Figure 2:**
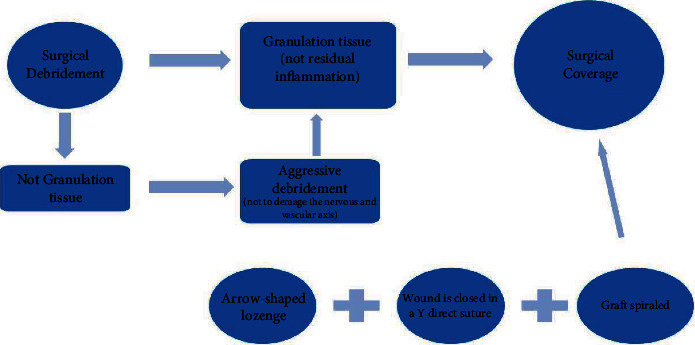
Schema of reconstruction treatment.

Extensive surgical debridement prevents the progression of FG. Aggressive debridement and local dressing changes should be done until granulation has been noted. A surgical coverage of the skin defect should be performed whenever the wound shows obvious signs of granulation, without any evidence of inflammation left (Figure 1(a). Exuberant granulation induces tissue reaction, and this may bury the penis, which will be more difficult to treat. The granulation tissue is revived with a curette or by scratching the tissue with a cold blade. During the procedure, it is important not to damage neither the nervous and vascular axis on the dorsal side of the penis nor the urethra on the ventral side. The remnants of the dartos and of the buck band are evaluated. The penis is pulled, and the skin defect is measured by length (L) and circumference (C). An arrow-shaped lozenge is drawn on the medial side of the arm pointing to the distal extremity to allow direct suture. The advantage of this donor site is its very thin skin. The length of the penis (L) presenting the loss of skin substance corresponds to the width of our arrow, while the circumference (C) is reported on the axis of the arrow (Figure 1(b)). The donor site is infiltrated with a solution of saline and adrenaline. The full-thickness skin graft is harvested with a cold blade and the wound is closed in a Y direct suture fashion. The graft is defatted, then pie-crusted to allow fluids and blood to drain adequately. In this arrow configuration, the tip is sutured to the bottom of the arrow, preventing a longitudinal scar contracture (Figure 1(c)). For smaller defects, a spindle-shaped graft is harvested from the medial side of the arm. The graft is then spiraled around the penis from the base to the extremity. To avoid wrinkles on the penis, the skin graft should be outstretched and tailored. The graft is then sutured with a simple interrupted resorbable suture and paraffin tulle dressing. Such interventions were performed following a rational scheme (Figure 2.)

## 3. Results

**Figure 3:**
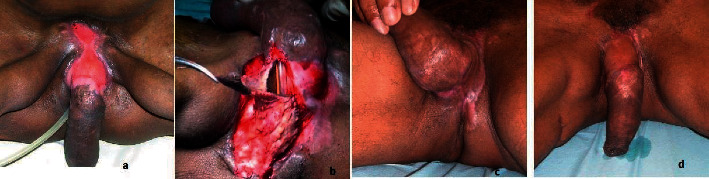
(a) Preoperative after positioning of the testicles on the internal side of the thigh before. (b) Intraoperative with a partial reconstruction of the urethra by local skin flap and full-thickness skin graft. (c-d) Result 1 year after.

**Figure 4:**
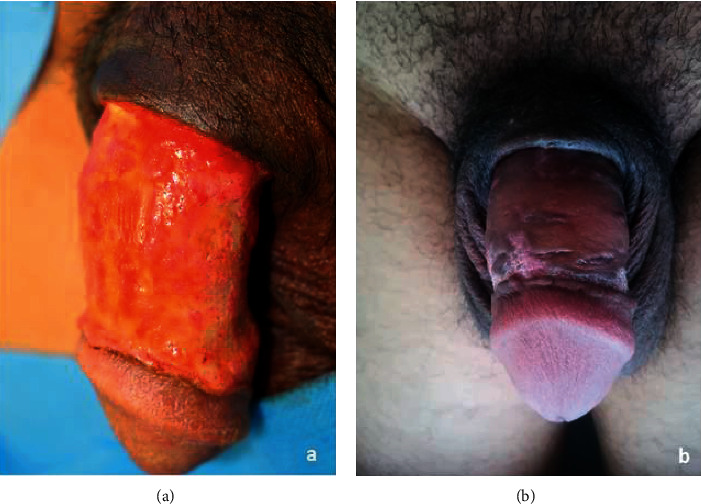
(a) Preoperative vision after debridement of the penis for local infection secondary to a subcutaneous injection of petrolatum. (b) 6 months postoperative result of a full-thickness skin graft for penile reconstruction.

From 2018 to 2021, 23 patients underwent surgical treatment for Fournier's gangrene of the penis (Table 1). Twelve patients (52%) presented a primary penis involvement of which 3 (11%) were due to penis trauma, 3 (11%) secondary to the self-injection of Vaseline, 2 (8.7%) complications of circumcision, 2 (8.7%) boil complications, 1 complication of epididymitis (4.3%), and 1 epidermoid carcinoma of the penis (4.3%). Eleven patients (48%) presented a secondary penis involvement after progressing perineal gangrene (Figures 3(a) and 3(b)). This unfavorable evolution was caused by: obesity and uncompensated diabetes in 4 patients (17%), perianal infection in 3 patients (11%), trauma injury in 3 patients (11%), and the onset of an infected sacral bedsore in a paraplegic patient. All our patients underwent an initial extensive debridement performed by a urologist to excise the infected and necrotic tissues. After granulation tissue developed, the reconstruction phase started (Figures 3(c) and 3(d)). In 12 cases the penile reconstruction with full-thickness skin graft was performed in one stage since the exclusive penis involvement (Figures 4(a) and 4(b)). In 11 cases debridement involved both the inguinal and scrotal regions, and priority was given to the reconstruction of these areas, while penis reconstruction was performed in a second stage. One patient whit important obesity (BMI 49) died due to a sepsis' aggravation. In 87% of cases (*n* = 20), no complications were observed. Partial lysis of the graft was reported in two cases (9%) and needed additional controlled wound healing. All patients were followed at 6 months and 1 year postoperative. No further penile revision surgery was required, and neither patient complained about retraction, nor traction, or pain during erection. The satisfaction of the esthetic and functional results was evaluated in patients after one year. They were asked to complete a questionnaire expressing their satisfaction in relation to the elasticity and comfort during erection, the shape and appearance of the penis, and the scar of the donor site using a Likert scale. Patients' opinions were rated on a 4-point scale including “poor,” “sufficient,” “good,” and “excellent” grades. 15 patients asserted “good” both for elasticity and comfort, and other 5 patients expressed “excellent” both for elasticity and comfort. No functional or psychological impotence was reported after reconstruction.

## 4. Discussion

**Figure 5:**
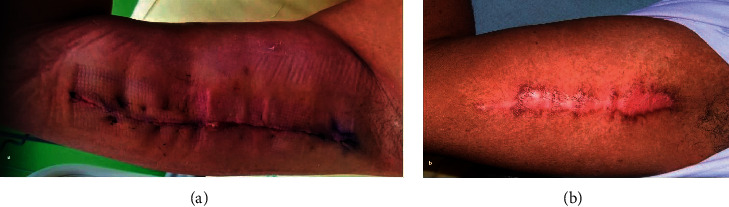
(a) Donator site immediately after the operation. (b) 6 months postoperative result.

Although the primary and isolated involvement of the penis in the gangrene of Fournier is considered rare [2, 3], the number of these cases (52%) in our data is comparable to the number of patients with secondary penile involvement in the context of larger gangrene (48%). Patients with secondary involvement were older and had more comorbidities, which may complicate the achievement of surgical outcomes. We performed a full-thickness skin graft for penile reconstruction in all cases. Vincent et al. in 1988 [16] reported superior cosmetic results with the use of full-thickness skin grafts over split-thickness skin grafts for penile defects. He asserted that FTSGs stretch with erection, have superior sensation long term, and have a less secondary contraction. The choice between full-thickness and partial-thickness skin grafts must balance different technical characteristics. Thin skin grafts have a greater grip and constitute ad almost infinite source of tissue while leaving a minimal harvesting scar. Thick grafts instead have greater coverage and a greater dermal component that guarantees a better esthetic result, but this kind of harvesting leads to a scar [17]. Anandan et al. asserted that either Split-thickness skin grafts or Full-thickness skin grafts may be used to reconstruct a buried penis either, depending on the preoperative evaluation performed by the surgeon [18, 19]. Split-thickness grafts are used more often in genital reconstruction since the graft survival rates are good, they lack hair follicles and there is no need for a local flap or subsequent grafting [20, 21]. In addition, these thin skin grafts would seem more like the penile skin which is thin and has truly little subcutaneous fat. However, in comparison, thick grafts tend to provide the best durability and show reduced rates of graft-related penile shrinkage, due to secondary contraction [18]. Demzik et al. [22] reported that partial and complete graft loss occurred in 8% of patients treated by STSG and in 3% of patients treated by FTSG. We propose for the first time the FTSG is harvested from the internal arm face instead of from the inguinal region [16, 23]. The medial face of the arm was carefully chosen as the donor site in all our cases (Figure 1(b)). This skin area is near to axillary fold and has a high waterproofness [24], a property that makes it less inclined to maceration. Moreover, this area lacks hair follicles compared to the inguinal region. In addition, FTSGs because of a greater thickness than STSGs are more elastic and resistant to traction, friction, and maceration, possible during the sexual act. In the cases shown, we found no complications related to the usage of full-thickness grafting. It also has comparable pliability and an excellent degree of elasticity as it is harvested in an extremely mobile area. The median face of the arm is an area where scars can be hidden with different techniques such as those adopted in the arm lift (Figure 5(a)). In fact, our arrow technique uses the arrow's tail to suture the donor site in a Y fashion, as described in the fish incision brachioplasty technique [25]. This way the proximal skin tension, as well as the risk of discomfort during movements, are both reduced **(**Figure 5(b)). Also, the arrow shape of the graft allows to easily orient it, to easily suture the tip to the bottom of the arrow. With our technique, we obtained a good esthetical outcome in the penile reconstruction secondary to Fournier's gangrene in all patients. No patient required further penile revision surgery. A delayed recovery was reported in only two cases. This type of complication is frequently observed in patients suffering from diabetes and obesity, which are known to be risk factors for delayed wound healing [26, 27]. We believe that to achieve an adequate penile reconstruction following Fournier's gangrene it is necessary that the exposed granulation tissue is sane and exempted from infectious necrotic residue [28–30]. It can be explained by the fact that very extensive gangrene needs longer local wound care to achieve an adequate infection-free state to receive the graft. Satisfaction of the esthetic and functional result was evaluated in 90% of patients with no report of functional or psychological impotence. The limit of our study is the lack of a statistical comparative study in terms of the rate between FTSGs and STSFs, we believe that it could be an interesting starting point for future insights.

## 5. Conclusion

We believe that our eleven years' experience in penile reconstruction with this grafting technique can be helpful to all operators, especially younger surgeons facing the difficult treatment of Fournier's gangrene with penile involvement. We believe that the coverage of the penis using our grafting technique is safe, easily reproducible, and demonstrates excellent esthetic and functional results.

## Figures and Tables

**Table 1:** Surgical management of our cases. ^*∗*^Likert scale (1 = Poor; 2 = Sufficient; 3 = Good; 4 = Excellent.

Cases	Age	Penis Involvement	Trigger Factor	Surgical Plan	Esthetic and functional result ^*∗*^
1	30	Primary	Self-injection of Vaseline	One-staged: full-thickness skin graft from the upper arm	3

2	42	Primary	Self-injection of Vaseline in a patient with Diabetes	One-staged: full-thickness graft from the upper arm	3

3	32	Primary	Self-injection of Vaseline	One-staged: full-thickness graft from the upper arm	3

4	2	Primary	Squamous cell carcinoma of the penis	One-staged: Full-thickness graft from the upper arm after resection	4

5	50	Primary	Epididymitis complication	One-staged: Burying of the testicles in a medial thigh pocket + full-thickness skin graft	3

6	48	Primary	Trauma injury	One-staged: full-thickness graft from the upper arm	3

7	59	Primary	Trauma injury	One-staged: full-thickness graft from the upper arm	4

8	25	Primary	Complication after circumcision	One-staged: full-thickness graft from the upper arm	4

9	50	Primary	Skin penis infection	One-staged: Scrotum reconstruction with scrotal residual flap + full-thickness skin graft from the upper arm	4

10	49	Primary	Skin penis infection	One-staged: Scrotum reconstruction with scrotal residual flap + full-thickness skin graft from the upper arm	3

11	20	Primary	Circumcision complication	One-staged: Urethra reconstruction and Full-thickness graft from the upper arm	3

12	65	Primary	Trauma injury	Two-staged:1. Burying of the testicles in a medial thigh pocket 2. Full-thickness skin graft from the upper arm	3

13	48	Secondary penile involvement in the context of pubic and scrotum gangrene.	The complication of Diabetes and Obesity	Two-staged 1. Burying of the testicles in a medial thigh pocket +split-thickness skin graft for perineal coverage 2. Full-thickness skin graft for the base of the rod	3

14	43	Secondary penile involvement in the context of perineal gangrene	The complication of Diabetes and Obesity	One-staged: Scrotum reconstruction with scrotal residual flap + full-thickness skin graft from the upper arm for penile reconstruction	4

15	50	Secondary penile involvement in the context of perianal gangrene	Perianal infection	Two-staged:1. Burying of the testicles in a medial thigh pocket 2. Full-thickness skin graft from the upper arm for penile reconstruction	4

16	29	Secondary penile involvement in the context of perianal gangrene	The complication of a sacral bedsore paraplegic patient	Two-staged: 1. Burying of the testicles in a medial thigh pocket 2. Full-thickness skin graft from the upper arm for penile reconstruction	3

17	52	Secondary penile involvement in the context of perineal Gangrene	Trauma injury	Two-staged: 1. Full-thickness graft for penile reconstruction2. Groin flap for scrotal reconstruction	3

18	59	Secondary penile involvement in the context of perineal gangrene	The complication of Diabetes and chronic renal failure	Two-staged: 1. Scrotum reconstruction with flaps 2. Full-thickness skin graft from the upper arm for penile reconstruction	3

19	58	Secondary penile involvement in the context of perineal gangrene	Perianal infection	Two-staged: 1. Burying of the testicle in a medial thigh pocket 2. Full-thickness graft for penile reconstruction	3

20	54	Secondary penile involvement in the context of perineal gangrene	Trauma Injury	Two-staged: 1. Scrotum reconstruction with scrotal residual flap + split-thickness graft for perineal coverage 2. Full-thickness skin graft from the upper arm for penile reconstruction	3

21	63	Secondary penile involvement in the context of perineal gangrene	Complication of obesity	Two-staged: 1. Burying of the remaining testicle in a medial thigh pocket2. Full-thickness graft from the upper arm for penile reconstruction	3

22	60	Secondary penile involvement in the context of perineal gangrene	Perianal infection	Two-staged:1. Burying of the testicles in a medial thigh pocket 2. Full-thickness skin graft from the upper arm for penile reconstruction	3

23	33	Secondary penile involvement in the context of perineal gangrene	Trauma injury	Two-staged: 1. Implantation of the testicles in a medial thigh pocket + full-thickness skin graft from the upper arm for penile reconstruction 2. Scrotum reconstruction with a groin flap	3

## Data Availability

The data used to support the findings of this study are available from the corresponding author upon request.
